# Combined detection of serum CTX‐II and C2C in a rat model of ACLT‐induced osteoarthritis

**DOI:** 10.1002/2211-5463.13613

**Published:** 2023-06-11

**Authors:** Fei Yu, Hui Bai, Zhiheng Zhang, TianWen Ma, Li Gao

**Affiliations:** ^1^ College of Veterinary Medicine Northeast Agricultural University Harbin China; ^2^ Heilongjiang Key Laboratory of Animals Disease Pathogenesis and Comparative Medicine Harbin China; ^3^ College of Veterinary Medicine Inner Mongolia Agricultural University Hohhot China

**Keywords:** biomarkers, C2C, CTX‐II, diagnosis, osteoarthritis, rat

## Abstract

Osteoarthritis (OA) is a chronic degenerative bone and joint disease that often occurs in aging animals. Currently, there are still no biomarkers that can effectively diagnose OA in the early stage. To identify possible biomarkers, here we examined changes in the expression of C‐telopeptide fragments of type II collagen (CTX‐II) and collagenase generated carboxy‐terminal neoepitope of type II collagen (C2C) in serum at different time points in an anterior cruciate ligament transection (ACLT)‐induced rat OA model. The serum levels of CTX‐II and C2C, and the OARSI score in the ACLT group were increased from week two until the end of the experiment. The AUC of the combined biomarkers was higher than that of CTX‐II or C2C alone. Moreover, serum levels of CTX‐II and C2C were positively correlated with the OARSI score. The results suggest that the combined detection of serum CTX‐II and C2C concentrations may have potential for assessing and diagnosing OA at early stages.

## Graphical abstract

Osteoarthritis (OA) is a degenerative joint disease that is difficult to diagnose in the early stages. The degradation of components of the extracellular matrix is a central mechanism of OA. CTX‐II and C2C, key fragments of type II collagen, are positively correlated with the OARSI score. Combined detection of CTX‐II and C2C may have potential for OA diagnosis.

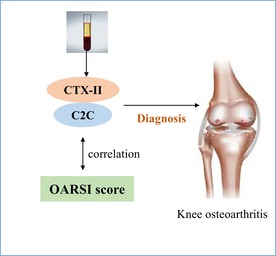

**Abbreviations**
ACLT: anterior cruciate ligament transection
C2C: collagenase generated carboxy‐terminal neoepitope of type II collagen
COMP: cartilage oligomeric matrix protein
CTX‐II: C‐telopeptide fragments of type II collagen
ECM: extracellular matrix
OA: osteoarthritis
OARSI: Osteoarthritis Research Society International
ROC: receiver operating characteristic curve

Osteoarthritis (OA) is a common joint diseases characterized by articular cartilage degeneration, synovitis, periarticular and subchondral bone changes [1]. OA is not only a wear process, but also involves the degradation of the extracellular matrix (ECM) [2]. Mechanical factors, inflammation, decreased muscle strength, joint damage, and obesity are common risk factors for OA [3]. A major concern of OA is the improvement of diagnostic tools and a more sensitive monitoring of the disease progression [4]. However, the value of the early diagnosis of OA is limited according to clinical symptoms [5]. Thus, several studies have demonstrated the potential of biomarkers of OA [6]. Blood‐based or urine‐based biomarkers are noninvasive and objective, and can be used for frequent monitoring of the disease activity.

The critical process of OA is the destruction of the articular cartilage and degradation of two major components of the ECM, including proteoglycans and type II collagen, making them markers of choice in evaluating cartilage metabolism [7]. The crosslinked CTX‐II [8] and C2C are potential biomarkers for predicting knee OA [9]. When assessed by magnetic resonance imaging (MRI), knee joint degeneration and OA are associated with serum levels of cartilage oligomeric matrix protein (COMP) and C2C [10]. Previous studies have shown that modulation of type II collagen degradative biomarkers such as C2C and CTX‐II are directly associated with major cartilage degradation turnover [11]. CTX‐II is highly correlated with the occurrence, development, injury degree, bone marrow lesion and osteophyte formation of OA [12]. For patients undergoing anterior cruciate ligament reconstruction, the level of CTX‐II was decreased with pain relief and physical function improvement, indicating a reduced disease severity [13].

Several recent research has suggested that analysis of type II collagen degradation, when used alone or in combination or with collagen synthesis markers, can indicate the progression of OA [14]. Therefore, our study aimed to explore the combination of CTX‐II and C2C in serum for OA diagnosis.

## Materials and methods

### Animals

Sixty male Sprague–Dawley rats (10 weeks old, 240–270 g) purchased from the Experimental Animal Management Center of Harbin Medical University were raised at the experimental animal center on a standard 12 h dark/light cycle (*n* = 6 per group). The ethical treatment of animals in this study was approved by the Animal Welfare Committee protocol (#NEAU‐2017‐02‐0252‐11) at Northeast Agricultural University (Harbin, China).

### Rat model of ACLT‐induced OA

All animals were adapted to the new environment 1 week before the test to reduce the stress response. Anterior cruciate ligament transection (ACLT) model and sham surgery was induced according to the previous protocols [15]. After anesthesia with isoflurane, the right knee joint skin was shaved. Next, the joint capsule was opened, the ACL was severed. Both capsule and skin were sutured using Vicryl 5‐0 (Ethicon, Edinburgh, UK) absorbable suture after the surgery. The serum samples and right knee samples in six independent groups (different animals in each group) were collected at 2, 4, 6, 8, and 10 weeks postoperatively. The knee samples were fixed with 4% paraformaldehyde for histopathology assessment. The serum was collected after centrifuged (1500 r.p.m., 15 min) and stored at −80 °C.

### Macroscopic observation

The degradation of cartilage on the surface of the tibial plateau was observed using a dissecting microscope. The morphology score was assessed according to the previous study [16]. Two observers blinded to the groups (*n* = 5) conducted macroscopic observations.

### Histopathology evaluation and OARSI score

After tissue fixative, the modified joints were placed in the embedding box and rinsed under running water for 24 h to remove excess fixative. Before decalcification, the tissue was put in a bucket filled with new decalcification solution (EDTA decalcification fluid; Servicebio, Wu Han, China) under a constant temperature shaker. The temperature and the rate of the shaker were generally controlled at 25–30 °C and 110–120 r.p.m., respectively. We use different concentrations of ethanol for dehydration. Then, xylene was soaked twice to replace the ethanol in the tissue, and the tissue without ethanol can be embedded in wax. The sample was placed in a bath filled with a paraffin solution at 65 °C for 150 min, and after curing, it was repaired into a trapezoidal shape for later use. After fixing the wax block and slicing, cut a slice with a thickness of 5 μm, pick it up and unfold it in warm water, spread it on a glass slide, and oven overnight. After Safranine O‐fast green staining, neutral gum was dropped on the specimen of the slide glass, and a cover glass was added to cover the slide. The score of articular cartilage destruction was based on the OARSI score system (*n* = 6) [17].

### ELISA

The serum of SD rats was thawed and the concentrations of CTX‐II and C2C in each group (*n* = 6) were assessed using ELISA kits according to the instructions of the manufacturer (catalog nos. EHJ‐96093r and EHJ‐96082r; Huijia Biological Technology Co., Ltd., Amoy, China).

### Statistical analysis

All data were analyzed using spss 22.0 software (IBM Corp., Armonk, NY, USA). One‐way ANOVA was performed and Tukey's honestly significant difference *post hoc* test for multiple comparisons. The *P*‐value < 0.05 was considered statistically significant. The correlation was analyzed using Spearman correlation analysis, and the sensitivity and specificity of marker detection were analyzed using ROC subjects' working characteristic curve. We used logarithmic transformation of some dependent variables to make the distribution more uniform. It made the hypothesis of normality and mean square deviation reasonable.

## Results

### Macroscopic examination of the joints in the ACLT‐induced OA model of rats

**Fig. 1:**
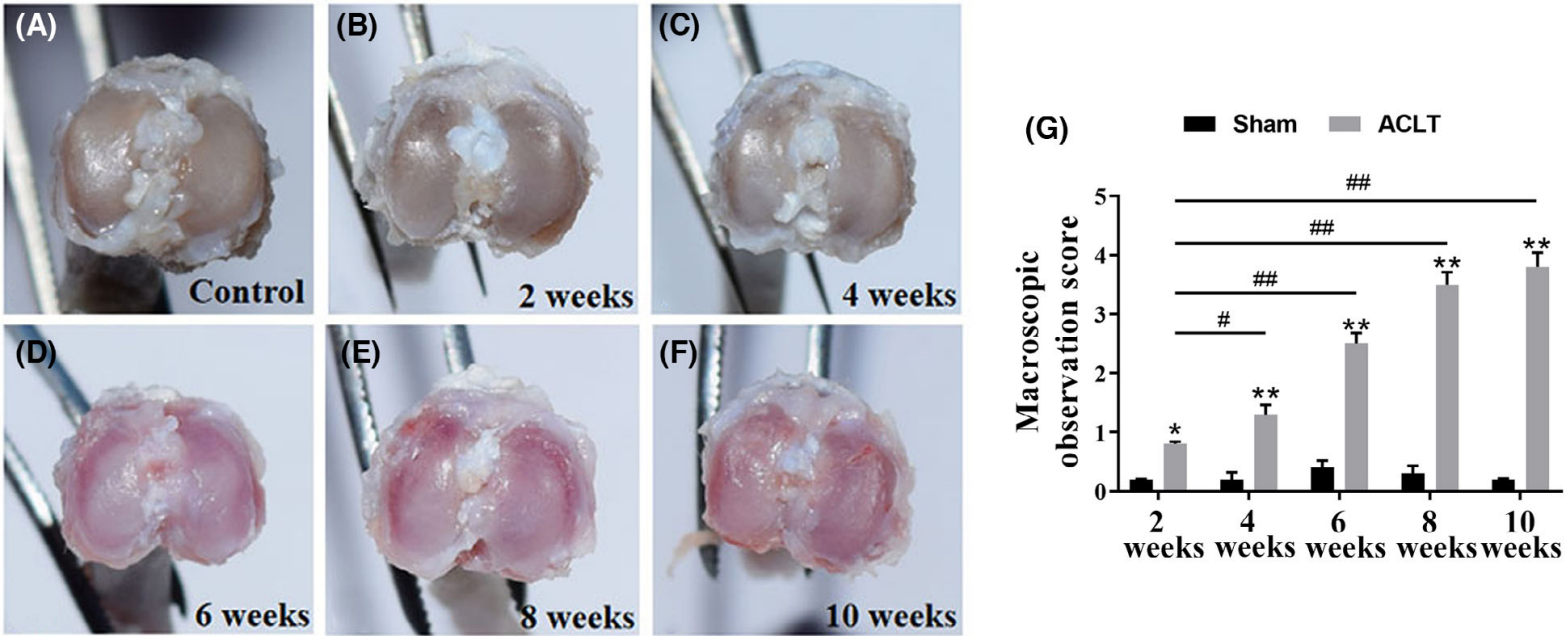
Macroscopic changes on knee joints. (A) Sham group; (B–F) ACLT group at week 2, 4, 6, 8, and 10 post surgery, respectively; (G) the gross morphology score (*n* = 5). *P < 0.05 vs. sham group, **P < 0.01 vs. sham group; ^#^ *P* < 0.05 vs. week 2 post ACLT surgery, *P* < 0.05; ^##^ *P* < 0.01 vs. week 2 post ACLT surgery. Values are the mean ± SD. One‐way ANOVA was performed and Tukey's honestly significant difference *post hoc* test for multiple comparisons.

A healthy articular cartilage was observed with smooth surface in the sham group (Fig. 1A). There were no obvious changes at week 2 following ACLT surgery (Fig. 1B). However, at week 4 after the surgery, we observed a mildly roughened surface with decreased transparency in the cartilage layer (Fig. 1C). Moreover, there were obvious macroscopic changes in the tibia at 6, 8 and 10 weeks post‐surgery. The cartilage was abraded (Fig. 1D) at week 6. Furthermore, the defect reached the deep layer of cartilage, and the subchondral bone could be seen in the same areas at week 8. The surface of the articular cartilage was worn severely and the cartilage became thinner (Fig. 1E). Finally, at week 10 after surgery, part of the cartilage was exfoliated and the subchondral bone was exposed. The roughness of the joint surface continued to increase, and apparent fissures appeared (Fig. 1F). In a word, the gross morphology of the tibia plateau of the right knee induced by ACLT surgery was severe gradually in a time‐dependent manner (Fig. 1G).

### Microscopic examination and OARSI score of the cartilage in the ACLT‐induced OA model of rats

**Fig. 2:**
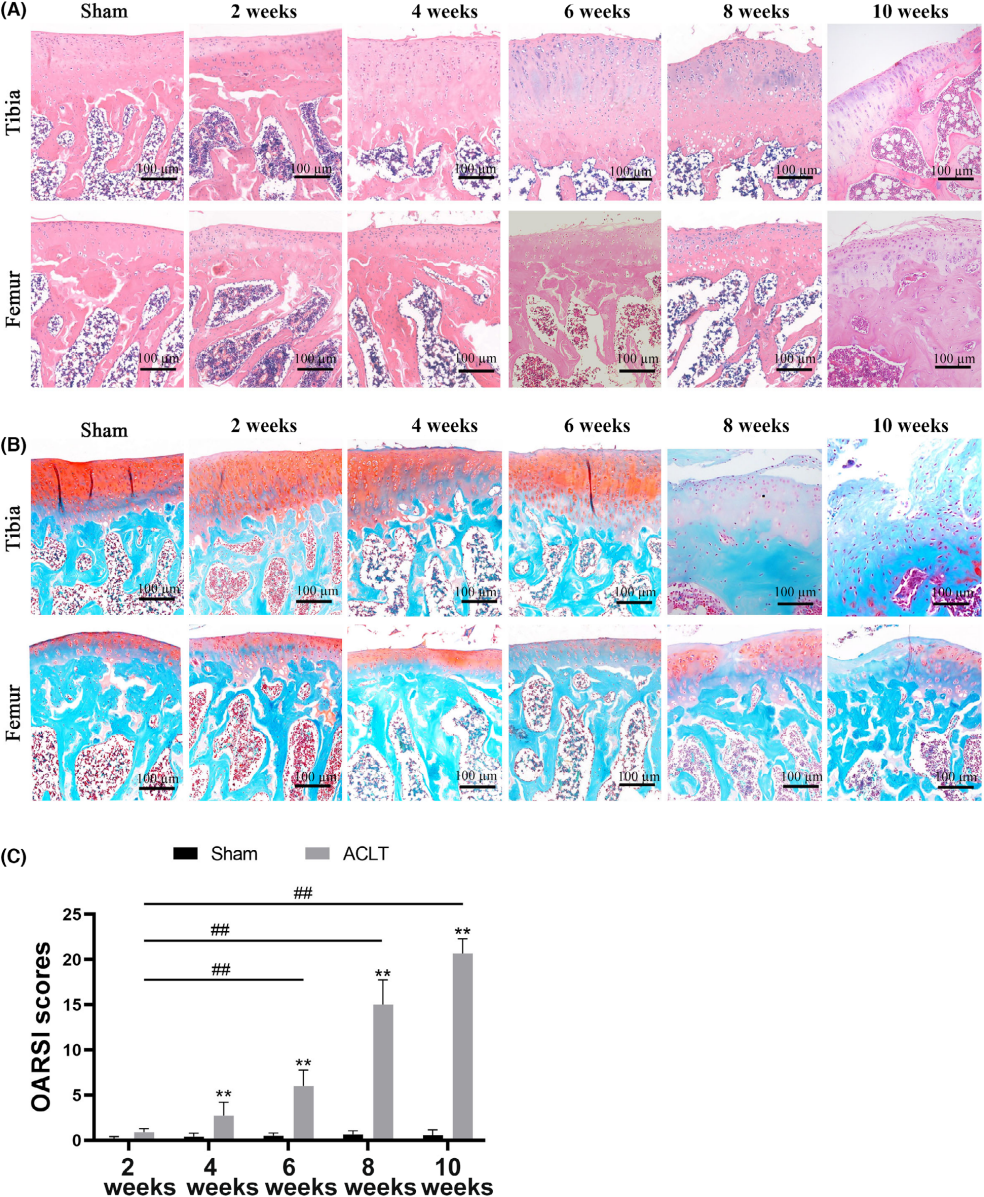
Histopathological changes and OARSI score of cartilage. (A) Representative images of cartilage by HE staining (*n* = 6). Scale bar: 100 μm. (B) Representative images of cartilage by Safranin O‐Fast Green staining (*n* = 6). Scale bar: 100 μm. (C) OARSI scores assessed by two experienced (*n* = 6) observers. **P < 0.01 vs. sham group; ^##^ *P* < 0.01 vs. week 2 post ACLT surgery. Values are the mean ± SD. One‐way ANOVA was performed and Tukey's honestly significant difference *post hoc* test for multiple comparisons.

We detected the severity of the ACLT‐induced OA model at different time points by assessing the integrity of cartilage. The sham group showed a smooth surface of cartilage and intact matrix, with no apparent changes (Fig. 2A,B). In contrast, the cartilage in ACLT‐induced rats showed slight injury 2 weeks post‐surgery, accompanied by superficial fibrillation and proliferation of chondrocytes in the superficial zone (characterized by chondrocyte clusters and/or disorientation of chondrocyte). However, the OARSI score showed no statistical significance compared to sham joints (Fig. 2C). Moreover, rats subjected to ACLT displayed surface discontinuity and chondrocytes clustering, and loss of orientation of the chondrons at week 4. At 6 weeks after ACLT surgery, the cartilage showed more severe damage compared to the ACLT‐induced joints at week 2, involving moderate to severe hypocellularity, a thickened perichondrium, and a reduction in Safranin‐O staining and increased OARSI score. In addition, an erosion occurred in the cartilage at week 8 post‐surgery, including delamination of the superficial layer (matrix cracks into the mid zone forming vertical fissures), loss of cartilage matrix, and cell death, as well as a higher OARSI score. Finally, we observed denudation in ACLT‐induced joints at week 10, which was characterized by complete erosion and denuded bone surface (Fig. 2A,B). The result was confirmed by OARSI scores. The severity of OA cartilage damage was aggravated in a time‐dependent manner with increased OARSI scores compared to the ACLT‐induced joints at week 2 (Fig. 2C).

### Changes of serum CTX‐II and C2C level in ACLT‐induced OA model of rats

**Fig. 3:**
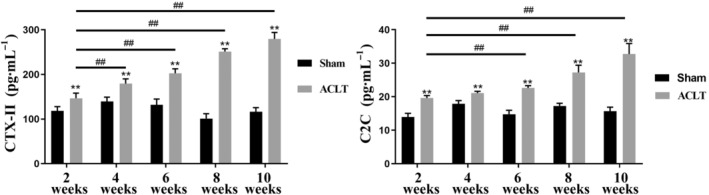
Changes of levels of CTX‐II and C2C in serum at different time points (*n* = 6). **P < 0.01 vs. sham group; ^##^ *P* < 0.01 vs. week 2 post ACLT surgery. Values are the mean ± SD. One‐way ANOVA was performed and Tukey's honestly significant difference *post hoc* test for multiple comparisons.

The serum concentration of CTX‐II and C2C in the sham and ACLT‐induced OA rats were detected using ELISA kits. As shown in Fig. 3, the concentrations of CTX‐II and C2C in the serum in the sham group showed no significant change, while the serum level of CTX‐II and C2C in the ACLT model were significantly increased compared to the sham group at weeks 2, 4, 6, 8 and 10 post‐surgery. Moreover, CTX‐II and C2C levels were significantly increased at weeks 6, 8 and 10 in ACLT joints as compared with ACLT joints at week 2.

### Correlation between serum levels of CTX‐II and C2C and OARSI score

**Fig. 4:**
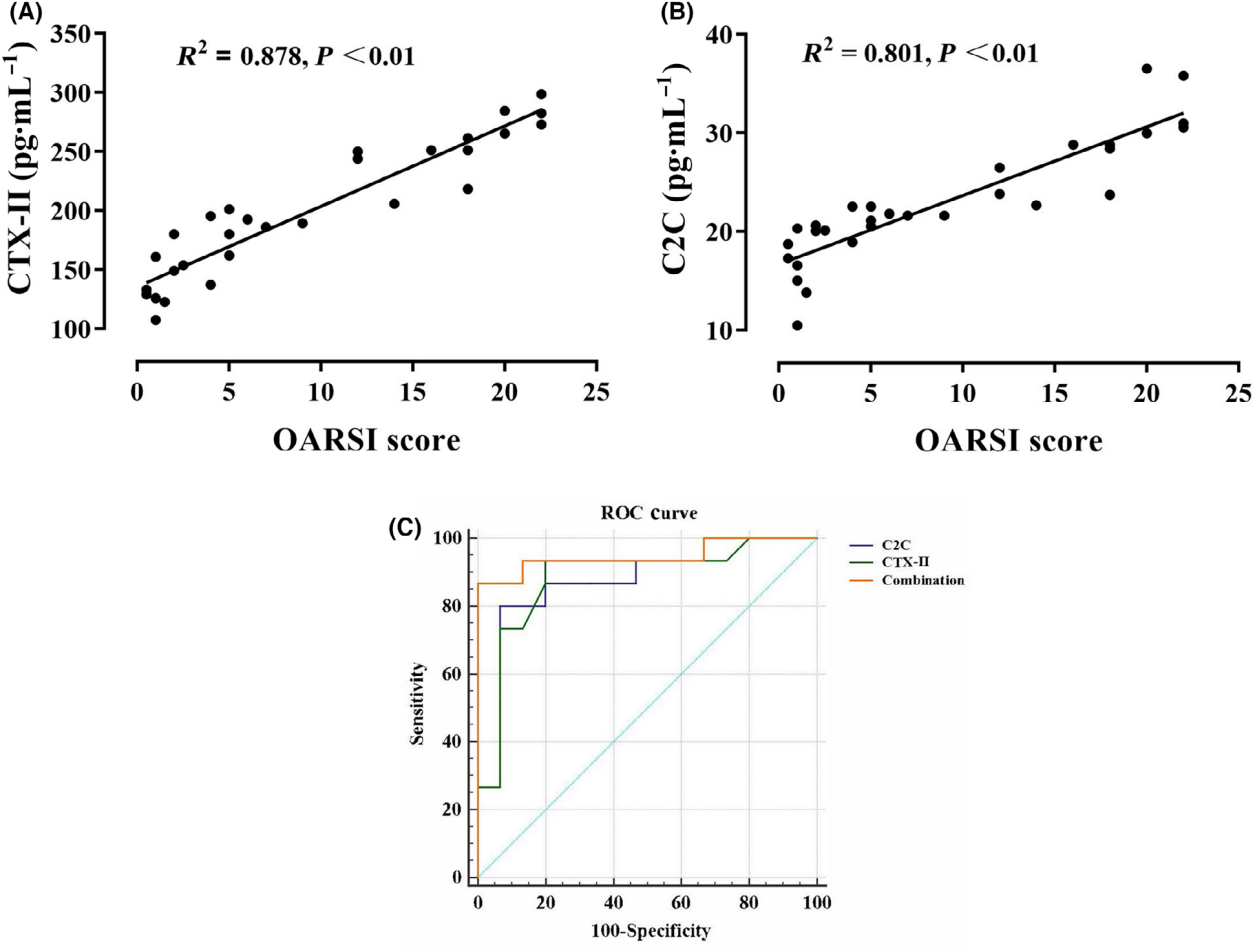
Correlation between serum CTX‐II and C2C levels and correlation with the OARSI score of articular cartilage. (A) Correlation between serum levels of CTX‐II and OARSI score (*r* = 0.916). (B) Correlation between serum levels of C2C and the OARSI score (*r* = 0.851). (C) ROC curve analysis of single and combined biomarkers in OA prediction. The diagonal segment is the reference line. The correlation was analyzed using Spearman correlation analysis, and the sensitivity and specificity of marker detection were analyzed using ROC subjects' working characteristic curve.

The Spearman correlation analysis showed a positive correlation between the serum level of CTX‐II and OARSI score in the ACLT group (*R* ^2^ = 0.878, *P* < 0.01) (Fig. 4A). In addition, the change in C2C concentration and OARSI score also showed a significant positive correlation (*R* ^2^ = 0.801, *P* < 0.01) (Fig. 4B).

### ROC analysis of CTX‐II, C2C, and combined biomarkers

The ROC analysis of the serum levels of C2C and CTX‐II showed that the areas under the curve (AUC) of C2C, CTX II, and the combined detection were more than 0.5, indicating that the single detection and combined detection of serum molecular markers have a specific diagnostic value for rat OA (Fig. 4C). The AUC values of CTX‐II, C2C, and combined detection were 0.882, 0.876 and 0.947, respectively. The data showed that the ROC value of the combined biomarker was significantly higher than that of a single biomarker.

## Discussion

Osteoarthritis is a common arthritis in veterinary medicine and poses considerable challenges to animal welfare. OA is well recognized as a significant clinical problem causing pain and disability in cats, dogs and horses [18, 19, 20]. In addition, equine OA is a significant cause of lameness and premature elimination of racehorses [21]. However, signs of OA are very subtle and unspecific in the early stage of the disease in clinical practice. Therefore, sensitive biomarkers that can be quickly detected in the early stage of the disease are crucial for predicting the occurrence and development of the disease. In addition, the diagnosis of animal OA is currently based on radiographic criteria and clinical symptoms, while the limitations of radiography (e.g., technical issues, precision and sensitivity) [22] promoted the development of biomarkers for monitoring OA. According to the BIPED classification, molecular biomarkers can be divided into the following five categories: disease severity markers, research markers, prognostic markers, therapeutic markers and diagnostic markers [23]. Both CTX‐II and C2C belong to tissue degradation markers and can be used as diagnostic markers in BIPED classification. The study results described the feasibility and sensitivity of CTX‐II and C2C in the combined detection of OA in rats, which is of high value for the early diagnosis of OA and can be used as comparative medical data to provide reference for the early diagnosis of OA.

Measurement of the degradation products of articular tissue, especially the activity of chondrocyte extracellular matrix has attracted much attention recently. CTX‐II and C2C are potentially predictive of OA. However, few studies that speculated that the combination of these two biomarkers has more potential for early OA diagnosis. In our present study, we investigated the articular cartilage degradation by measurement of serum CTX‐II and C2C levels in a 10‐week longitudinal study using an ACLT surgical rat model. The results indicated that the OA severity was aggravated, and the serum levels of CTX‐II and C2C were increased in a time‐dependent manner. In addition, the ROC curve diagram suggested that a combination of CTX‐II and C2C would be ideal for diagnosing OA.

Various animal models are available to mimic human OA progression, including intra‐articular injection of a chemical drug, surgery‐induced joint instability, and a high‐fat diet‐induced obesity model [24]. Among those models, ACLT‐induced surgery showed apparent advantages, specifically rapid progression and low cost [25]. The advantages of ACLT model was that the cartilage changes in ACLT model were closer to the developmental state of OA disease, and this model was suitable for relevant studies on drug screening for OA treatment [26]. Most of our previous studies have used this model [27], and we have skilled surgical techniques and a thorough understanding of the occurrence and development of OA in this model, which can ensure the repeatability and stability of the model. The present study investigated the macroscopic and microscopic changes of articular cartilage at different time points. The changes in morphology and histology of joints were significantly deteriorative following ACLT surgery, including loss of matrix, erosion of deep layer of cartilage, and cell death. The results were consistent with a previous study, which demonstrated OA changes developed progressively with time in ACLT‐induced OA in rats [26].

Osteoarthritis is a common arthritis, characterized by degradation of extracellular matrix (ECM) and chondrocyte apoptosis, causing dyskinesia and disability in elderly patients [28]. Type II collagen and proteoglycans are two primary components of ECM. As highly sensitive molecules, biomarkers have been widely studied in OA [29, 30, 31, 32]. CTX‐II, a C‐telopeptide of type II collagen, changes significantly during OA development and progression. Naito *et al*. [33] reported that CTX‐II increased significantly with cartilage histological damage following mechanical OA. Most studies have shown the predictive value of urinary levels of CTX‐II for the loss of cartilage [34]. A recent study reported the clinical value of serum CTX‐II measurement in ACLT‐induced rabbits [35]. The results of this study showed that CTX‐II changes in young rabbits and adult rabbits were different. The serum level of CTX‐II in adult rabbits increased significantly during the first 3 weeks following the surgery, followed by a decrease until week 6 and a second rise from week 7 to week 12. From the peak of the week 12, a decrease was observed until the end of the study. However, the serum level of CTX‐II in young rabbits just increased until week 2, and then decreased all along the study until the end [35]. However, our findings suggested otherwise. Our results showed that the concentration of CTX‐II in ACLT group increased significantly as compared with the sham group. Moreover, the serum level of CTX‐II increased continuously all along the study until week 10 post‐surgery. Of note is the rats used in our study were 10‐weeks‐old. Thus, they were young rats. The different trends of CTX‐II levels between rabbits and rats might be attribute to the species differences. It is important to note that rabbits generally do not reach bone maturity until they are 8–9 months of age [36, 37], and rabbit cartilage exhibits spontaneous healing, particularly in young animals up to 20 weeks of age [38, 39], suggesting the decreased levels of CTX‐II in young rabbits in Duclos ME's study might due to spontaneous healing.

Type II collagen is continuously degraded and cleaved into two length fragments by matrix metalloproteinases during OA development. The exposure of a new epitope in a fragment with a length of 3–4 is called C2C. Various studies described the association between C2C in urine and radiographic progression of knee OA [40]. Interestingly, the serum C2C level is correlated with knee degeneration in patients with symptomatic knee OA [41]. In our study, the concentration of C2C in the model group increased significantly compared to the control group.

In order to improve diagnostic sensitivity and accuracy, we determined the histopathological examination. We observed positive correlations between serum CTX‐II or C2C and OARSI score. The histological results in our study showed that the articular cartilage has been damaged from week two following ACLT surgery and aggravated with time. Our results suggested that CTX‐II and C2C might be suitable biomarkers for a longitudinal study [42]. Previous studies reported that the metabolic cycle of type II collagen in normal cartilage is very long, and the concentration of its metabolic degradation product CTX‐II can evaluate the degradation of type II collagen for a certain period. Further, it can reflect the degree of degradation of type II collagen in mineralized tissues [43]. In a follow‐up study, Cahue found that C2C levels were closely related to the disease severity [44]. Other studies have shown that the CTX‐II level strongly correlated with the microscopic severity score of joint disease in the collagen‐induced rat OA model [45]. We conducted the Spearman analysis on the changing trend of serum CTX‐II and C2C concentrations and OARSI score in the rat OA model. The results showed a significant correlation between CTX‐II, C2C, and the OARSI score, indicating that the changing trend of CTX‐II and C2C concentrations is closely related to the severity of OA. Therefore, CTX‐II and C2C might be effective biomarkers for diagnosing of early OA.

Osteoarthritis is heterogeneous with a variety of phenotypes. Although our findings showed that C2C and CTX‐II were highly correlated with OA progression, they still have limitations. We used the ACLT‐induced rat model to mimic post‐traumatic OA, while the availability of these biomarkers in other OA phenotypes need further exploration, including obesity‐induced OA and age‐related OA. In our present study, the serum levels of C2C and CTX‐II were increased from week two after ACLT surgery and showed a time‐dependent tendency. Accordingly, OARSI scores showed similar changes. However, severe cartilage destruction occurred from week 8, attributed to the gradual development of ACLT‐induced OA models [26]. Further, biomarkers can detect in the early stage of OA, which was helpful for diagnosing OA early. Our study found that the increase in C2C and CTX‐II occurred from week two onwards, which was consistent with previous study [46].

The change of CTX‐II and C2C in the serum has a high correlation with the severity of mechanical OA and might be a potential combined biomarker for diagnosing OA, which is more stable and sensitive than a single biomarker. However, this study has some limitations, and the longitudinal research cycle is insufficient and the clinical symptoms such as pain and motor dysfunction should take in consideration. Further studies should assess CTX‐II and C2C changes for a longer duration and the clinical symptoms of OA need further examination.

## Conclusions

The concentrations of CTX‐II and C2C in the serum of OA rats increased with the severity of cartilage injury, and severity of cartilage injury was increased with time, moreover there was a positive correlation between them. The degree of cartilage injury is positively correlated with the corresponding OARSI score. The ROC value of the combined biomarker was higher than that of a single biomarker. The combined diagnosis of CTX‐II and C2C biomarkers of mechanically induced OA has predictive value for identifying the severity and early diagnosis of OA.

## Conflict of interest

The authors declare no conflict of interest.

## Author contributions

The experiments were designed by ZZ and LG. The experiments were performed by ZZ, HB and TM. FY collected and analyzed data. FY wrote and edited the manuscript. HB revised the manuscript. All authors read it and finally approve the final version.

## Data Availability

All data generated or analyzed during this study are included in the published article and its Supporting Information files.
